# Stable *STIM1* Knockdown in Self-Renewing Human Neural Precursors Promotes Premature Neural Differentiation

**DOI:** 10.3389/fnmol.2018.00178

**Published:** 2018-06-11

**Authors:** Renjitha Gopurappilly, Bipan Kumar Deb, Pragnya Chakraborty, Gaiti Hasan

**Affiliations:** National Centre for Biological Sciences, Tata Institute of Fundamental Research, Bengaluru, India

**Keywords:** calcium signaling, SOCE, inducible shRNA-miR, RNA-Seq, neural development

## Abstract

Ca^2+^ signaling plays a significant role in the development of the vertebrate nervous system where it regulates neurite growth as well as synapse and neurotransmitter specification. Elucidating the role of Ca^2+^ signaling in mammalian neuronal development has been largely restricted to either small animal models or primary cultures. Here we derived human neural precursor cells (NPCs) from human embryonic stem cells to understand the functional significance of a less understood arm of calcium signaling, Store-operated Ca^2+^ entry or SOCE, in neuronal development. Human NPCs exhibited robust SOCE, which was significantly attenuated by expression of a stable shRNA-miR targeted toward the SOCE molecule, STIM1. Along with the plasma membrane channel Orai, STIM is an essential component of SOCE in many cell types, where it regulates gene expression. Therefore, we measured global gene expression in human NPCs with and without *STIM1* knockdown. Interestingly, pathways down-regulated through *STIM1* knockdown were related to cell proliferation and DNA replication processes, whereas post-synaptic signaling was identified as an up-regulated process. To understand the functional significance of these gene expression changes we measured the self-renewal capacity of NPCs with *STIM1* knockdown. The *STIM1* knockdown NPCs demonstrated significantly reduced neurosphere size and number as well as precocious spontaneous differentiation toward the neuronal lineage, as compared to control cells. These findings demonstrate that *STIM1* mediated SOCE in human NPCs regulates gene expression changes, that *in vivo* are likely to physiologically modulate the self-renewal and differentiation of NPCs.

## Introduction

The advent of pluripotent stem cells in the past decade, which includes embryonic stem cells (ESC) and induced pluripotent stem cells (iPSCs) and their neural derivatives, has allowed direct cellular and molecular analysis of human cell-derived brain progenitors as well as differentiated neurons. This is in contrast to earlier studies where understanding the biology and function of human brain cells was largely restricted to post-mortem and pathological specimens (Di Lullo and Kriegstein, 2017). From cellular studies in other organisms it is evident that Ca^2+^ signaling affects a range of neural activities during development including neurotransmitter specification (Marek et al., 2010 ; Spitzer, 2012 ; Plazas et al., 2013 ; Guemez-Gamboa et al., 2014), synaptogenesis and neurite extension (Rusanescu et al., 1995 ; Rosenberg and Spitzer, 2011 ; Kawamoto et al., 2012). Besides well-documented modes of Ca^2+^ entry in neuronal cells through ligand and voltage-gated Ca^2+^ channels, it is evident that Endoplasmic Reticulum-driven store-operated Ca^2+^ entry (SOCE), also functions in neurons (Bardo et al., 2006). Store-operated Ca^2+^ entry (SOCE) is based on the interaction of STIM proteins, that sense Ca^2+^ levels in the Endoplasmic Reticulum (ER; Liou et al., 2005 ; Roos et al., 2005), with the Orai Ca^2+^ channel in the plasma membrane and was first described in non-excitable cells (Feske et al., 2006 ; Vig et al., 2006 ; Zhang et al., 2006). More recently it has also been characterized in excitable cells (Venkiteswaran and Hasan, 2009 ; Hartmann et al., 2014 ; Lalonde et al., 2014 ; Pathak et al., 2015) where significant roles for intracellular Ca^2+^ stores, and potentially SOCE, have been suggested in neurogenesis and neural development (Toth et al., 2016). Neuronal SOCE has been recognized as an important mechanism that neurons use to replenish Ca^2+^ stores during cell activation. As in non-excitable cells, SOCE might also initiate specific signaling pathways in NPCs and differentiated neurons (Majewski and Kuznicki, 2015).

SOCE through Orai channels has been investigated in mouse NPCs, isolated from embryos where it regulates gene transcription through calcineurin/NFAT (nuclear factor of T cells) signaling (Somasundaram et al., 2014). Interestingly, the attenuation of SOCE in mouse NPCs reduced their proliferation *in vitro*, as well as in the sub-ventricular zone (SVZ) of adult mouse brains *in vivo* (Somasundaram et al., 2014). Moreover, a recent study demonstrated that pharmacological blockade of SOCE in mouse SVZ neural stem cells decreased proliferation and impaired self-renewal by shifting the type of SVZ stem cell division from symmetric proliferative to asymmetric (Domenichini et al., 2018). Although mechanisms of neurogenesis are largely conserved among mammals, human neurogenesis shows some distinct attributes when compared to that of rodents, such as the presence of an astrocyte ribbon in the sub-ventricular zone (SVZ), higher turnover of hippocampal neurons and a rapid decline in the rate of neurogenesis with age (Sanai et al., 2004 ; Knoth et al., 2010 ; Wang et al., 2011). In the adult rodent brain NSCs continue to give rise to new neurons (van Praag et al., 2002 ; Toni et al., 2008), whereas adult neurogenesis in humans is still controversial (Boldrini et al., 2018 ; Sorrells et al., 2018). Rates of cell growth and neural lineage differentiation between primary rodent and human NSCs/NPCs are also reported to be dissimilar (Ostenfeld et al., 2002). Human stem cell-derived NPCs exhibit greater variation in neurite outgrowths when compared to rat cortical cultures (Harrill et al., 2011). *In vitro*, human NPC lines and mouse-derived cell lines respond differently to multiple stimuli, including *trans* -retinoic acid known to affect proliferation and induce apoptosis (Culbreth et al., 2012). Furthermore, differences in cell surface markers (Klassen et al., 2001) and shorter telomeres have been demonstrated in hNPCs (Ostenfeld et al., 2000) as compared to their rodent counterparts. Independent studies in human neural precursors and differentiated neurons are thus essential to understand how STIM/Orai based SOCE might impact human brain development and function.

To understand the functional significance of SOCE-regulated gene expression in human neural cell specification we generated human NPCs (hNPCs) from a human embryonic stem cell line. The hNPCs exhibit robust SOCE that was significantly attenuated by knockdown of *STIM1*. Global transcriptomic analysis of *STIM1* knockdown hNPCs revealed downregulation of pathways associated with cell proliferation and concomitantly an upregulation of genes for neural differentiation. These changes in gene expression correlate with reduced proliferation and early neural differentiation in *STIM1* knockdown hNPC cultures indicating that the loss of SOCE *in vivo* could result in cessation of sufficient hNPCs, required for normal brain development.

## Materials and Methods

### Experimental Design

We hypothesized that abrogating store-operated Ca^2+^ entry (SOCE), the less studied arm of endoplasmic reticular (ER) centric Ca^2+^ homeostasis mechanisms, in human neural precursors (hNPCs), may lead to disruption of their normal function based on studies of various neuronal cell types in rodents (reviewed by Toth et al., 2016) and *Drosophila* (Pathak et al., 2015, 2017 ; Richhariya et al., 2017). We generated small molecule induced hNPCs from a well-characterized human embryonic stem cell line (hESCs) (Reinhardt et al., 2013) and successfully knocked down *STIM1*, an essential element of SOCE, through lentiviral transduction to obtain expandable stable *STIM1* knockdown hNPC lines. Ca^2+^ imaging and immunoblots confirmed *STIM1* knockdown and the attenuation of SOCE. To investigate cellular and molecular changes brought about by loss of SOCE RNAseq analyses of the *STIM1* knockdown NPCs and their appropriate vector controls were performed that helped to identify significant changes in gene expression. Changes in expression levels of selected genes, identified by RNAseq, were further validated by real-time PCR. To understand the functional significance of SOCE-regulated changes in gene expression, Gene Ontology analyses were performed and a set of enriched biological pathways were identified that underwent significant up or downregulation. The identified pathways helped design experiments for phenotypic and functional characterization of the *STIM1* knockdown NPCs. Such experiments based on the identified GO pathways, corroborated a cell fate change in *STIM1* knockdown NPCs (**Supplementary Figure S1**). Details of the bioinformatics analyses, statistical tests performed and methods for the wet lab experiments are provided in the following segments.

### Maintenance and Neural Induction of Human Embryonic Stem Cells (hESCs)

All experiments, performed with hESC lines, were approved by the Institutional Committee for Stem Cell Research, registered under the National Apex Committee for Stem Cell Research and Therapy, Indian Council of Medical Research, Ministry of Health, New Delhi. The hESC cell line H9/WA09 (RRID: CVCL_9773) was used for this study. Undifferentiated hESCs were initially cultured on irradiated mouse embryonic fibroblasts and gradually adapted to grow under feeder-free conditions by culturing on 0.5% Matrigel in complete mTeSR media (Stem Cell Technologies, Vancouver, BC, Canada). Passage of cells was initiated by washing with phosphate-buffered saline (PBS) followed by incubation at 37°C in CTK dissociation solution (PBS with 0.25% trypsin, 1 mg/mL collagenase IV, 20% KSR, all from Invitrogen, Carlsbad, CA, United States and 1 mM CaCl_2_ from Sigma, St Louis, MO, United States). hESC cultures were allowed to form embryoid bodies (EBs) by forced aggregation in low attachment dishes. For neural induction, as described earlier (Reinhardt et al., 2013 ; Louis et al., 2017) 2-day EBs, were supplemented with 10 mM SB431542 (Stem Cell Technologies), 1 mM dorsomorphin (Tocris Cookson, Ballwin, MO, United States), 3 mM CHIR99021 (Stem Cell Technologies) and 0.5 mM purmorphamine in suspension cultures. Four-day EBs were treated with 1:1 DMEM/F12 neurobasal medium supplemented with 1:200 N2, 1:100 B27 along with neural induction media factors in suspension cultures. Six-day old EBs were plated onto Matrigel-coated plates in maintenance medium containing 1:1 DMEM/F12 neurobasal medium supplemented with 1:200 N2, 1:100 B27, 3 μM CHIR99021, 0.5 mM purmorphamine and 150 μM ascorbic acid (Sigma, St Louis, MO, United States). Neural precursor cells (NPCs) were then passaged enzymatically with Accutase (Invitrogen) and stored frozen in liquid nitrogen. Batches of frozen NPCs were thawed and plated as per requirement (protocol adapted from Reinhardt et al., 2013). NPCs could be maintained for >25 passages, though for all experiments described here we used cells from passage numbers of less than 25. For spontaneous differentiation, neural precursors were allowed to grow in media, without small molecules and in the presence of N2 and B27 supplements, for 14–21 days. Media was replenished every alternate day for NPCs and for spontaneously differentiating cultures.

### shRNA-miRs and Lentiviral Transduction for *STIM1* Knockdown

ShERWOOD-UltramiR short hairpin RNA (shRNA), are vector-based RNAi triggers with a new generation shRNA-specific design and an optimized microRNA scaffold “UltramiR” (Auyeung et al., 2013 ; Knott et al., 2014). *STIM1* knock-down was performed using a mixture of *STIM1-* ULTRA-3374033 *(TAATATTGCACCTCCACCTCAT)*, ULTRA-3374029 (*TTTATGATCTACATCATCCAGG*) and ULTRA-3374031 (*TCCAGTGAGTGGATGCCAGGGT*) (transOMIC Technologies, Huntsville) in NPCs. A Non-Targeting shRNA Construct (NTC) was used as a control for all experiments. The inducible ZIP (all-in-one) vector contains the components necessary for regulated expression of the shRNA-mir, including the TRE3GS inducible promoter positioned upstream of the shRNA, and the Tet-On 3G transcriptional activator (Tet-On 3G TA), which is expressed constitutively from an internal promoter. The Tet-On 3G TA binds to the TRE3GS promoter in the presence of doxycycline and induces expression of ZsGreen and the shRNA-mir. This allows for direct visual confirmation of induced shRNA expression. A puromycin resistance gene (PuroR) is also encoded for rapid selection of transduced cells. The lentiviral transfer vector (pZIP) was co-transfected with the desired packaging vectors (pCMV-dR8.2 and pCMV-VSVG from Addgene RRID: SCR_002037) encoding the *env, gag* and *pol* protein into a packaging cell line (HEK293T- ATCC Cat# CRL-3216, RRID: CVCL_0063). The transfer vector contained sequences that package as the viral genome. The sequences encode an shRNA-mir against *STIM1* and a selection cassette, both of which integrate into the target cell’s genome. Viral particles, released from the packaging cell, were harvested from the supernatant for 3 days as follows. Virus-containing supernatant was filtered through 0.45 μm PVDF syringe filters (Millipore), concentrated using a Lenti-X-concentrator, tested with Lenti-X GoStix (Clontech) and applied to NPCs (P-10) at an MOI of 10. After 24 hrs the media surrounding the NPCs was discarded and fresh media with doxycycline was added to induce shRNA expression. Cells were maintained in doxycycline containing media for a minimum of 5 passages to obtain an NPC cell line with stable knockdown. Cell counts and growth curves of the NTC and *STIM1* KD cells were obtained by the Trypan Blue exclusion method. The MTT [3-(4,5-dimethylthiazol-2-yl)-2,5-diphenyltetrazolium bromide, Sigma] assay for proliferation was performed as described earlier (Burke and Kukoly, 2008).

### Ca^2+^-Imaging in hNPCs

Ca^2+^-measurements were performed on hNPCs plated as single cells on poly-D -lysine (PDL) coated coverslips. After plating, cells were allowed to attach for 24 h prior to imaging. Cells were washed thrice with the culture medium, following which they were loaded with 5 μM of the ratiometric Ca^2+^-indicator Indo-1 acetoxymethylester (AM, Invitrogen,) which was dissolved in the culture medium and supplemented with 0.002% Pluronic F-127 in dark for 45 min. Following this, the cells were washed three times with the culture medium and kept in it until imaging. Just before the start of imaging, the culture medium was replaced with ‘0 Ca^2+^ HBSS’ (20 mM HEPES, 137 mM NaCl, 5 mM KCl, 2 mM MgCl_2_, pH = 7.3) supplemented with 0.5 mM EGTA and 10 mM Glucose. To check for inhibition of SOCE, inhibitors of the SOC-channel, BTP-2 (Invitrogen) and 2-APB (Invitrogen) or DMSO (solvent control) were dissolved in the indicated concentration in 100 μl of the ‘0 Ca^2+^ HBSS.’ For these experiments, the culture medium in which the cells were kept after washing was replaced by HBSS containing either the inhibitors or DMSO and imaged. Therefore, the SOCE-inhibitors were present in the external medium throughout the course of the imaging. Ca^2+^ changes were recorded from single cells using a 60X oil objective (NA = 1.35) and 350-nm excitation and 405/485 dual-emission filter sets in an Olympus IX81-ZDC2 Focus Drift Compensating Inverted Microscope. Basal changes in cytosolic Ca^2+^ in ‘0 Ca^2+^ HBSS’ were recorded every 5s for 10 frames, after which Thapsigargin (TG, 10 μM, Invitrogen) was added to the cells to induce ER store Ca^2+^ release. After acquiring cytosolic Ca^2+^ changes every 15 s for 30 frames, 2 mM CaCl_2_ was added to the cells to induce SOCE, which was also recorded every 15 s for 30 frames. 10 μM Ionomycin (Calbiochem) was finally added to record the maximum fluorescence values obtained after saturating the dye with Ca^2+^. Image acquisition was performed using the Andor iXON 897E EMCCD camera and AndoriQ 2.4.2 imaging software (RRID: SCR_014461). The time-lapse acquisition mode of the software was used to follow fluorescence changes over time. A region of interest (ROI) was drawn manually around each cell. Fluorescence intensities of single cells were computed with ImageJ (RRID: SCR_003070) at 405 and 485 nm and these were used to calculate the F405/485 ratio for each time point. The F405/485 indicates the ratio of the fluorescence intensity emitted by the ‘Ca^2+^-bound’ to the ‘Ca^2+^-unbound’ form of the dye in the cells at each time point. Ca^2+^ responses from several cells were averaged and the mean (+ SEM) across different time points was used to represent Ca^2+^-responses during store-release and SOCE. Peak F405/485 values were quantified as box-plots, where the box represents the spread between 25 and 75% of the data, using Origin 8.0 software (RRID: SCR_014212). Horizontal lines in the center of the box represent the median and the smaller squares represent the mean. Statistical significance was computed among data sets using Mann–Whitney *U* test after applying the Bonferroni correction. Unless otherwise mentioned, all chemicals were obtained from Sigma.

Quantification of basal cytosolic [Ca^2+^] from hNPCs was performed using the dual-excitation single emission ratiometric Ca^2+^-indicator Fura-2-AM. hNPCs plated as single adherent cells on PDL-coated coverslips were washed thrice with culture medium, following which they were loaded with 5 μM Fura-2-AM, in the dark, for 45 min at room temperature. The dye was dissolved in culture medium supplemented with 0.002% Pluronic F-127. After dye loading, cells were washed thrice with culture medium. The culture medium was finally replaced with HBSS containing 2 mM Ca^2+^ (20 mM HEPES, 137 mM NaCl, 5 mM KCl, 10 mM Glucose, 1 mM MgCl_2_, 2 mM CaCl_2_, pH = 7.3). Fura-2 was excited using dual 340/380 nm excitation and the emission intensity was recorded at 510 nm. Basal changes in cytosolic Ca^2+^ were recorded for 10 frames at an interval of 5 s. To obtain the minimum fluorescence values, 10 mM EGTA was added for chelating all available cytosolic Ca^2+^, following which fluorescence changes were recorded every 5 s for 85 frames. Subsequently, the extracellular medium was supplemented with 10 mM Ca^2+^. The maximum fluorescence intensities were recorded after saturating the dye loaded within the cell with Ca^2+^, by adding 10 μM Ionomycin. Images were acquired after Ionomycin addition for 20 frames at 5 s interval. The peak fluorescence value was generally obtained within the first 2 frames (corresponding to 10 s) of Ionomycin addition. Emission intensities corresponding to excitation at 340 and 380 nm were used to calculate the F340/380 ratio for each cell across all time points. The basal F340/380 at the start of imaging (*t* = 0) was calibrated to [Ca^2+^] using the Grynkiewicz equation (Grynkiewicz et al., 1985) as follows:

$$\left[{Ca}^{2+}\right]\left(nM\right)=K_{d}X\beta X\left(R-R_{\min}\right)/\left(R_{\max}-R\right),$$

where R_min_ and R_max_ correspond to the minimum F340/380 and maximum F340/380 obtained after EGTA and Ionomycin addition, respectively. K_d_ for Fura-2 in human cells was taken as ∼225 nM (Forostyak et al., 2013) and the scaling factor β = 5, was based on the ratio of the fluorescence emission intensities of Ca^2+^-free and Ca^2+^-bound forms of the dye after excitation at 380 nm. Basal cytosolic [Ca^2+^] values calculated as described above, for NTC and *STIM1* knockdown cells, were quantified as box plots. Quantification was from a minimum of 50 cells for all genotypes/treatment (except the wild-type untreated cells, *n* = 25) from 3 plates of each type. Statistical significance was computed using Mann–Whitney *U* test.

### Immunocytochemistry and Quantitative Image Analysis

hESC-derived NPCs or spontaneously differentiating progenitors were fixed with 4% paraformaldehyde, permeabilized in 0.1% Triton X-100 and blocked with 4% FBS. The cells were incubated with primary antibodies overnight at 4°C, following which they were washed and incubated with fluorescent-labeled secondary antibodies for 2 h at 37°C, washed again and finally covered with 60% glycerol (v/v) for imaging with an Olympus IX73 (Olympus Corporation, Shinjuku, Tokyo, Japan) inverted microscope using 10X and 20X objective lenses. Images were analyzed and quantified using the Q-Capture Pro (RRID: SCR_014432) and ImageJ (RRID: SCR _003070) software, respectively. For confocal microscopy, an inverted Olympus FV1000 confocal microscope was used with a 60X oil objective, the Fluoview 2.1 C software (Olympus Fluoview FV10-ASW, RRID: SCR_014215) and FV10-SPD detectors, under optimal settings. Cell nuclei were counterstained with 300nM DAPI (4, 6-diamidino-2-phenylindole, Sigma) and 10–15 random fields from 3 to 4 plates were acquired for each immunostained sample. Student’s unpaired *t* -test, assuming unequal variance, was used for *p* -value calculation from three independent experiments. Details of the primary antibodies used are as follows:

STIM1,1:500 (Cell Signaling Technology Cat# 5668S, RRID: AB_10828699); Sox2, 1:150 (Abcam Cat# ab97959, RRID: AB_2341193); Tuj1, 1:1000 (Promega Cat# G7121, RRID: AB_430874); Sox1, 1:500 (Abcam Cat# ab87775, RRID: AB_2616563); Nestin, 1:500 (Abcam Cat# ab92391, RRID: AB_10561437); MAP2, 1:500 (Abcam Cat# ab32454, RRID: AB_776174); Doublecortin, 1:500 (Abcam Cat# ab18723, RRID: AB_732011); VIMENTIN, 1:500 (BD Biosciences Cat# 550513, RRID: AB_393716); TH, 1:2000 (Millipore Cat# AB152, RRID: AB_390204); Ki-67, 1:400 (Millipore Cat# AB9260, RRID: AB_2142366).

### Library Preparation, Sequencing and RNA-Seq Data Analysis

Total RNA was isolated from hNPCs using TRIzol as per manufacturer’s instructions. The RNA was run on a Bio-analyzer chip (Agilent) to ensure integrity. Approximately 500 ng of total RNA was used per sample to prepare libraries (RIN values > 9) using a TruSeq RNA Library Prep Kit v2 (Illumina) following manufacturer’s instructions. The prepared libraries were run on a DNA1000 chip of a Bio-analyzer to check their size. Libraries were then quantified by qPCR and run on an Illumina Hiseq2500 platform, for a single end and 75 bp read protocol (SciGenom, India). Nine samples were run in a single lane. Biological triplicates were performed for each sample consisting of RNA isolated from wild-type NPCs, shRNA control NPCs (referred to as the Non-Targeting Control or NTC) and *STIM1* knockdown NPCs.

More than 100 million reads were obtained per sample with a uniform distribution of reads across samples (**Figure 3B**). FASTQ sequencing reads obtained were aligned to the annotated UCSC human genome (GRCh37/hg19) using HISAT2, RRID: SCR_015530 (Version-2.0.5) (Kim et al., 2015). These aligned SAM files were converted to BAM files using SAMtools (Version- 1.3) (Li et al., 2009). The resulting alignment data were then fed to CuffDiff2, RRID: SCR_001647, a software package that takes the reads aligned in BAM format as input, and uses geometric normalization on gene-length normalized read counts (FPKM, fragments per kilo base of exon per million reads), a beta negative binomial model for distribution of reads and *t* -test, for calling differentially expressed genes (Trapnell et al., 2012 ; Seyednasrollah et al., 2015). We set a corrected *p* -value, referred to as the *q* -value cut-off of 0.05 and Fold change > / = 1.5(+/-) to identify differentially expressed genes (DEGs) by this method. Read counts for each transcript or exon were also calculated independently using the Python based package HTSeq (Version 0.9.1) (Anders et al., 2015). These read counts were used as inputs for differential analysis with DESeq, RRID: SCR_000154 (Anders et al., 2015) and EdgeR (Empirical analysis of digital gene expression in R), RRID: SCR_012802 (Anders and Huber, 2010 ; Robinson et al., 2010), two R based Bioconductor software packages that analyze the read counts per transcript per sample and normalize them using the method of Trimmed Mean of *M* -values (TMM) for removing genes with very low read counts. DEGs were identified by fitting the values obtained in a negative binomial model, with variance and mean linked by local regression (Anders and Huber, 2010 ; Robinson et al., 2010 ; Lin et al., 2016). A fold change of 1.5 (+/-) and a *p* -value of 0.05 in DESeq and an FDR *p* -value of 0.05 were set as the cut-off in EdgeR. Genes found to be significantly altered by all the three differential gene expression analysis methods were considered further. Significantly up and downregulated genes were separately subjected to a gene ontology based gene enrichment analysis tool, DAVID (Version 6.8) (Database for Annotation, Visualization and Integrated Discovery) (Dennis et al., 2003) and FunRich (Functional enrichment analysis tool) (Pathan et al., 2015), using the human genome as the background gene set. After converting the input gene IDs to corresponding DAVID gene IDs, the Functional Annotation Tool was used for gene enrichment analysis, based on the DAVID knowledge base. Fisher’s exact *p* -value method was employed to measure gene-enrichment in DAVID, with a *P* -value cut-off of 0.1 and the count threshold kept at 2 to speculate maximum information. Most significant biological pathways (GO level 5) enriched in DAVID have been reported as bar graphs. Gene enrichment analysis was also performed using the human Gene Ontology database, HPRD2 and FunRich (Pathan et al., 2015). Here, selected biological processes were identified based on the presence of a higher percentage of genes (>6%). Genes enriched in each identified pathway have been represented as heat maps based on their FPKM values. The density box plot and dendrogram were generated using CummeRbund, RRID: SCR_014568 (Goff et al., 2012). Heat maps were generated using Matrix2png, RRID: SCR_011614 (Pavlidis and Noble, 2003) and HemI (Heatmap Illustrator, Version 1.0.3.7) (Deng et al., 2014). Comparison of significantly altered gene lists from CuffDiff, DESeq and EdgeR, and the generation of Venn Diagrams were performed using FunRich. The data discussed in this publication have been deposited in NCBI’s Gene Expression Omnibus (Edgar et al., 2002) and are accessible through GEO Series accession number GSE109111.

### Quantitative Real-Time PCR

RNA was isolated from cells using TRIzol as per manufacturer’s instructions. Quantity of the isolated RNA was estimated by a NanoDrop spectrophotometer (Thermo Scientific). Approximately 1 μg of total RNA was used per sample for cDNA synthesis. Three or more independently isolated RNA samples were tested for validation of gene expression by quantitative PCR. Total RNA was treated with 0.5U of DNase I (amplification grade) in a reaction mixture (22.1 μl) containing 1 mM DTT and 20U of RNase inhibitor. The reaction mixture was kept at 37°C for 30 min followed by heat inactivation at 70°C for 10 min. To this, 200U of MMLV reverse transcriptase, 50 μM random hexamers, and 1 mM dNTPs were added in a final volume of 25 μl for cDNA synthesis. The reaction mixture was kept at 25°C for 10 min, then 42°C for 60 min, and finally heat inactivated at 70°C for 10 min. Quantitative real-time PCRs (qPCRs) were performed in a total volume of 10 μl with Kapa SYBR Fast qPCR kit (KAPA Biosystems) on an ABI 7500 fast machine operated with ABI 7500 software (Applied Biosystems). Duplicates were performed for each qPCR reaction. GAPDH was used as the internal control. The fold change of gene expression in any experimental condition relative to wild-type was calculated as 2^-ΔΔCt^, where ΔΔCt = (Ct_(target gene)_ - Ct_(GAPDH)_) from *STIM1* knockdown cDNA – (Ct_(target gene)_ - Ct_(GAPDH)_) from NTC cDNA. Four independent samples in addition to the samples used for the RNA-Seq were quantified for each gene. Statistical significance was determined by the unequal variance *t* -test. Primer sequences (F, forward primer and R, reverse primer) for each gene tested by qPCR are given below:

**Table:** 

*GAPDH*	F-TCACCAGGGCTGCTTTTAACTC
	R-ATGACAAGCTTCCCGTTCTCAG
*STIM1*	F-CACACTCTTTGGCACCTTCC
	R-TGACAATCTGGAAGCCACAG
*UNC5C*	F-ACGATGAGGAAAGGTCTGCG
	R-AAGTCATCATCTTGGGCGGC
*ELAVL3*	F-CAAGATCACAGGGCAGAGC
	R-ACGTACAGGTTAGCATCCCG
*DLG4*	F-ACCAAGATGAAGACACGCCC
	R-CCTGCAACTCATATCCTGGGG
*NFAT4*	F-CCGTAGTCAAGCTCCTAGGC
	R-TCTTGCCTGTGATACGGTGC
*LIN28A*	F-AAGAAGTCAGCCAAGGGTCTG
	R-CACAGTTGTAGCACCTGTCTC
*BAX*	F-CGGGGTTTCATCCAGGATCG
	R-CGGCAATCATCCTCTGCAGC


### Western Blots

For total protein quantification, cells were lysed using a 1X cell lysis buffer (Cell Signaling Technologies) with protease inhibitors and phosphatase inhibitors as per manufacturer’s protocol. Protein quantity was estimated by the Bradford assay and an equal amount of protein was loaded for immunoblotting. Proteins were transferred onto nitrocellulose membranes. The blots were blocked with 5% skimmed milk or bovine serum albumin for an hour at 37°C. After blocking, the blot was incubated with a primary antibody overnight at 4°C and probed with the respective secondary antibody conjugated to horseradish peroxidase (HRP), by incubation for an hour at 37°C. Antibodies for STIM1 and DCX were used at 1:1000 dilutions, Tuj1 at 1:2000, Sox2 at 1:300 (RRIDs provided in the section on immunocytochemistry above), and Actin (Loading control) at 1:3000 (BD Biosciences Cat# 612656, RRID: AB_2289199). Secondary antibodies used were anti-mouse HRP (1:3000; Cell Signaling Technology Cat# 7076, RRID: AB_330924) and anti-rabbit HRP (1:3000; Thermo Fisher Scientific Cat# 32260, RRID: AB_1965959). Bands were visualized with a chemiluminescence kit (GE Healthcare, Little Chalfont, United Kingdom) and captured using a chemiluminescent detection system (ECL, Thermo Scientific) with ImageQuant software (RRID: SCR_014246). Blots have been cropped for presentation in the main figures. Original images of the blots used are shown in **Supplementary Figure S3**. Unequal variance *t* -test was performed on band densities, obtained after background-subtraction, from at least three independent experimental blots.

### Neurosphere Formation Assay

H9-derived NPCs, both control (NTC) and *STIM1* knockdown cells, were seeded into 96-well plates at a density of 50 cells/μl per well. Fresh medium was added every other day, and spheres were counted after 1 week. The total numbers and the average size of neurospheres were counted from bright-field images of each independent condition performed as replicates.

### Statistical Analyses

For comparison between two samples, a two-tailed unpaired Student’s *t* -test was used and the *p* -value is stated in the respective figures and figure legends. All statistical tests were performed using Origin 8.0 software (RRID: SCR_014212). Mann–Whitney *U* test after applying the Bonferroni correction was used for the Ca^2+^ Imaging data. The Fischer exact *P* -value was used for RNA-Seq data analysis, after Benjamini–Hochberg procedure and Bonferroni correction. Statistical tests are also explained in the respective method sections. All data are pooled from three or more independent experiments and are presented as mean ± SEM. Asterisks indicate (^∗^) *p* ≤ 0.05, (^∗∗^) *p* ≤ 0.01 and (^∗∗∗^) *p* ≤ 0.001. Final figures were made in Adobe Photoshop (RRID: SCR_014199) or Inkscape (RRID: SCR_014479).

## Results

### Derivation of Expandable Neural Precursor Cells (NPC) From Human Embryonic Stem Cells (hESC)

**FIGURE 1:**
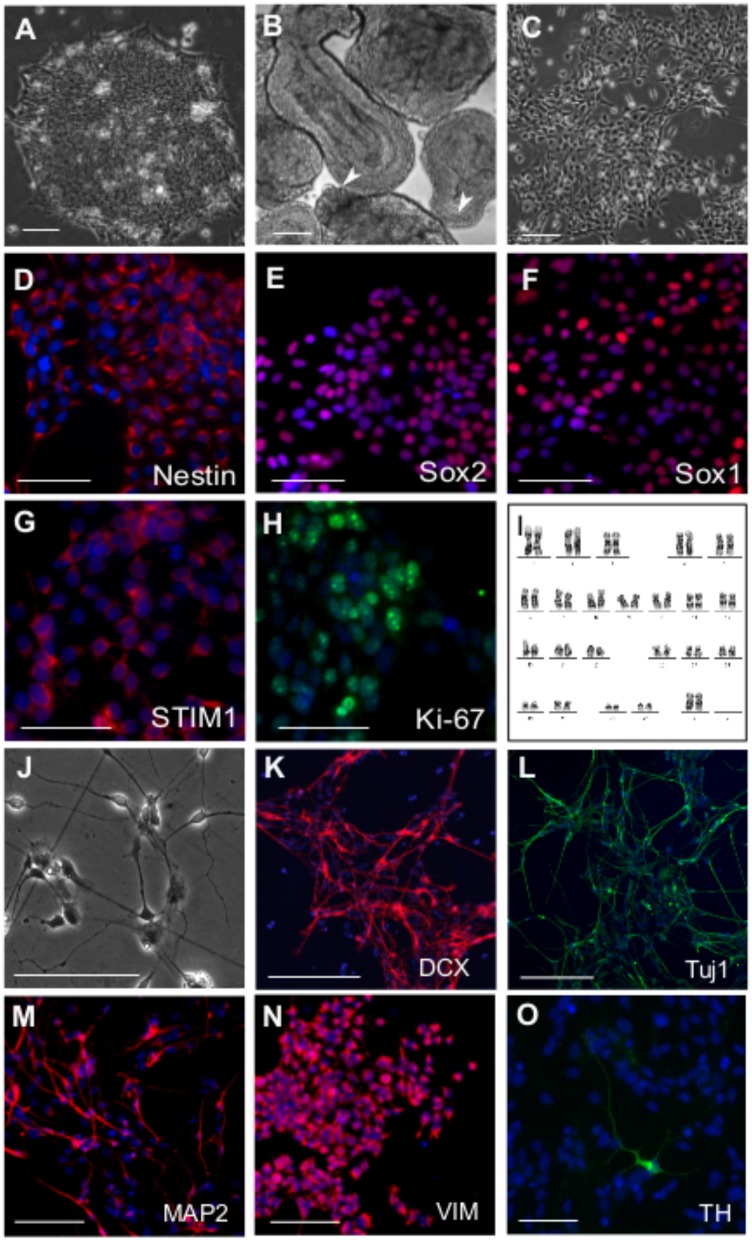
Derivation of neural precursor cells (NPC) from hESC. Phase contrast images of **(A)** hESC colony grown on matrigel **(B)** Day 4 EBs showing epithelial outgrowths (white arrowheads) when grown in the presence CHIR99021, a GSK 3β inhibitor and Purmorphamine, an activator of Shh pathway. **(C)** Neural precursor cells (NPCs) at passage 5, three days after split. Immunostaining of NPCs with antibodies raised against the neural stem/precursor cell markers as indicated **(D)** Nestin **(E)** Sox1 **(F)** Sox2. NPCs showing robust expression of **(G)** STIM1 protein, the ER calcium sensor and **(H)** Ki-67, a proliferation marker. **(I)** Karyogram of NPCs at passage 10 showing a normal karyotype (XX). Differentiation of NPCs into neural derivatives where cells were allowed to spontaneously differentiate for 10–14 days, **(J)** Phase contrast image of a day 12 spontaneously differentiating NPC culture, immunostained for the neuronal markers **(K)** Dcx **(L)** Tuj1 **(M)** MAP2 and the astroglial progenitor marker **(N)** Vimentin. **(O)** TH positive dopaminergic neuron after 21 days in culture. Nuclei are counterstained with DAPI in all immunostaining panels. Scale bars are 100 μm **(A–H)** and 50 μm **(K–O)**. Representative images are from 2 to 4 independent experiments.

Neural differentiation of human Embryonic Stem Cells (hESCs) relies on an enhanced understanding of developmental signaling mechanisms that drive neurogenesis in a physiological context (Yap et al., 2015). The use of small molecules to successfully induce neural differentiation and to generate neural precursors *in vitro* has been established reproducibly in multiple cases (Efe and Ding, 2011 ; Li et al., 2011 ; Chambers et al., 2012 ; Neely et al., 2012). We derived neural precursors from hESCs (**Figure 1A**) by a dual SMAD inhibition protocol where rapid neuroectoderm differentiation was induced by inhibition of BMP and TGFβ signaling (Reinhardt et al., 2013). To maintain the developmental potential for both neural crest and neural plate, Shh signaling was inhibited in the differentiating embryoid bodies (EBs) and a GSK3β inhibitor was included to stimulate canonical Wnt signaling (Reinhardt et al., 2013). EBs in suspension exhibited epithelial outgrowths (**Figure 1B**) and formed homogeneous colonies when plated on Matrigel after disaggregation. Neuro-epithelial cells (**Figure 1C**) thus derived could be passaged and maintained for more than 25 passages without any changes in cell kinetics. The cells expressed biomarkers of neural precursors such as Nestin, Sox1, and Sox2 (**Figures 1D–F**). Robust expression of the STIM1 (**Figure 1G**) protein (but not STIM2, data not shown) was also observed along with the proliferation antigen Ki-67 (**Figure 1H**). Because the NPCs were not derived by rosette-based manual selection (Zhang et al., 2001 ; Elkabetz et al., 2008), chromosome stability of P10 metaphasic NPCs was analyzed. The karyotype appeared normal (**Figure 1I**) indicating that small molecule and enzymatic treatment used for deriving the NPCs did not incur chromosomal aberrations. The cells exhibited good post-thaw recovery rates after cryopreservation. Their cell-growth kinetics and marker expression profiles were reproducible after several freeze-thaw cycles and from different passages (data not shown). Upon withdrawal of the small molecules from the media, rapid neural differentiation was seen as evident from expression of neuroblast and post-mitotic neuronal markers DCX (Doublecortin), Tuj1 (Neuron-specific Class III β-tubulin) and MAP2 (Microtubule Associated Protein-2) in neuronal progenitors (**Figures 1J–M**), along with astroglial progenitors marked by Vimentin (**Figure 1N**). However, in agreement with previous findings astroglia-like cells appeared much later (at least after 7 days of differentiation) than their neuronal counterparts and were restricted to smaller areas in the culture dish (Qian et al., 2000). Interestingly, such spontaneous differentiation also yielded a few dopaminergic neurons as indicated by immunostaining with an antibody to Tyrosine Hydroxylase (TH) (**Figure 1O**).

### SOCE in hNPCs and Its Attenuation With *STIM1* shRNA-miR

**FIGURE 2:**
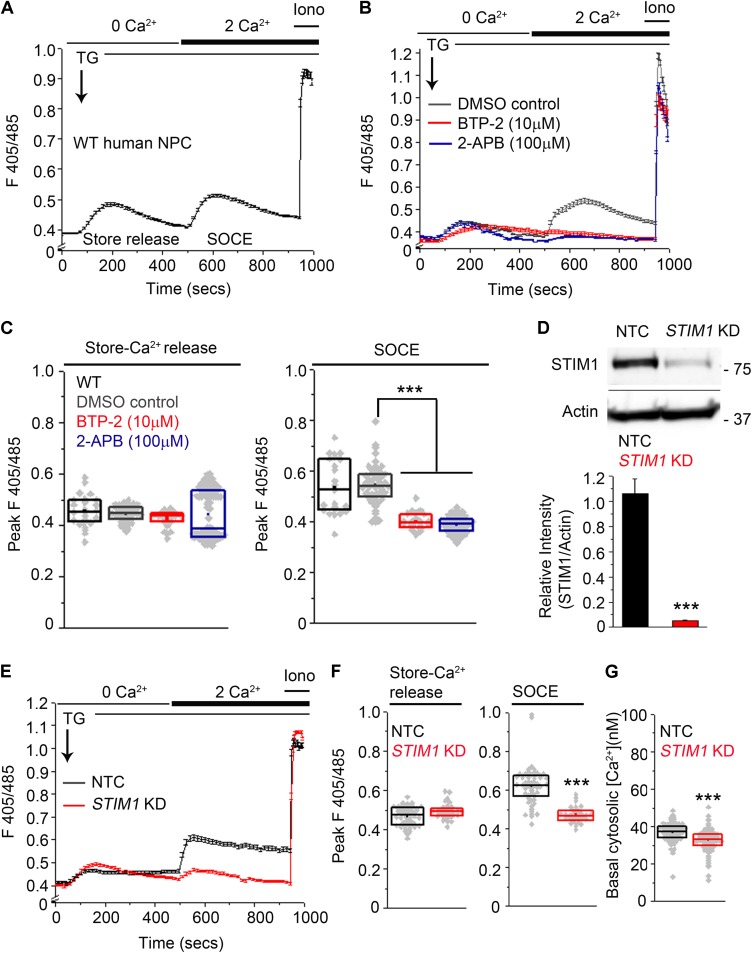
Knockdown of *STIM1* attenuates SOCE in human NPCs. **(A,B)** Ca^2+^-responses during ER-store release and SOCE induced by Thapsigargin (TG, 10 μM) measured using the ratiometric Ca^2+^-indicator indo-1-AM in wild-type (WT) hNPCs **(A)** or hNPCs treated with pharmacological inhibitors of SOCE, BTP-2 and 2-APB at the indicated concentrations or DMSO as a solvent control **(B)**. Each trace represents the mean ±SEM for 25–100 cells. Ionomycin (Iono, 10 μM) was added at the end of each imaging to determine the peak F405/485 ratio obtained after saturation of the Ca^2+^-indicator with Ca^2+^. **(C)** Box plots quantifying the peak F405/485 values for store-release and SOCE in the indicated treatment conditions. Mann–Whitney *U* test with Bonferroni correction. *p* = 1.819 × 10^-23^ for DMSO control compared to BTP-2 treatment and *p* = 1.442 × 10^-45^ for DMSO control compared to 2-APB treatment. **(D)** (Top) A representative Western blot showing levels of *STIM1* protein in hNPCs transduced with an NTC (non-targeting control) or an sh-RNA targeting *STIM1* (*STIM1* KD). Actin serves as the loading control. (Bottom) Quantification of STIM1 band intensities normalized to the loading control Actin from three independent biological replicates (*p* = 0.00069, Student’s *t* -test). **(E)** Ca^2+^-responses during store-release and SOCE in hNPCs transduced with NTC and *STIM1* KD. **(F)** Box plots quantifying the peak F405/485 values for store-release and SOCE in the indicated genotypes. Peak F405/485 for store-release were not significantly different between NTC and *STIM1* KD NPCs. *p* = 0.0001 for peak F405/485 during SOCE compared between NTC- and *STIM1* KD NPCs. **(G)** Quantification of basal cytosolic [Ca^2+^] values using Fura-2-AM in NTC- and *STIM1* KD NPCs (*p* = 1.115 × 10^-8^. Mann–Whitney *U* test. (^∗∗∗^Indicates *p* < 0.001).

To determine whether small molecule-derived NPCs exhibit SOCE, we depleted ER stores using 10 μM thapsigargin (TG), an inhibitor of the sarcoendoplasmic reticulum Ca^2+^ATPase pump (Lytton et al., 1991) in a Ca^2+^-free solution and studied Ca^2+^ influx after re-addition of extracellular 2 mM Ca^2+^. ER-store Ca^2+^ release, followed by SOCE after re-addition of external Ca^2+^ was observed consistently across several passages in human NPCs (**Figure 2A**). CRAC (calcium release-activated calcium) channels, identified as Orai1, and distinguished by high Ca^2+^ selectivity and a unique pharmacological profile (Feske et al., 2006 ; Lewis, 2011 ; McNally and Prakriya, 2012 ; Jairaman and Prakriya, 2013 ; Prakriya and Lewis, 2015) function in mouse NPCs as Store-Operated Calcium entry channels (Somasundaram et al., 2014). Therefore, we tested if potent CRAC channel inhibitors like BTP-2 (Ohga et al., 2008) and 2-aminoethoxy-diphenyl borate (2-APB, Prakriya and Lewis, 2001) affect SOCE in human NPCs. Both BTP-2 (Bootman et al., 2002) and 2-APB significantly inhibited SOCE in human NPCs (**Figures 2B,C**). Thus, the pharmacological profile of SOCE in human NPCs is consistent with that of CRAC channels and resembles SOCE in primary mouse NPCs (Somasundaram et al., 2014 ; Prakriya and Lewis, 2015).

In order to knockdown *STIM1* expression, NPCs (P10-P12) were transduced with *STIM1* shRNA lentiviral particles (Auyeung et al., 2013 ; Knott et al., 2014). Direct visual confirmation of induced shRNA expression through ZsGreen and rapid selection of transduced NPCs with Puro^R^ enabled efficient propagation of the transduced cells. Human NPCs transduced with a non-targeting vector control (NTC) were used as controls for all subsequent experiments. Western blot analyses confirmed maximal *STIM1* knockdown (>90%, *p* = 0.00067) in NPCs transduced with a pool of three *STIM1* targeting shRNAs (**Figure 2D** and **Supplementary Figure S3A**). Subsequent experiments were performed with NPCs at P18-P22. NPCs with *STIM1* knockdown (henceforth referred to as *STIM1* knockdown) exhibited a significant reduction in SOCE as compared to the corresponding control, whereas release of store Ca^2+^, after inhibition of the sarcoplasmic ER Ca^2+^-ATPase by thapsigargin treatment, appeared similar to control cells (**Figures 2E,F**). The mean basal cytosolic calcium levels in control and *STIM1* knockdown cells were 38 and 32 nM respectively (**Figure 2G**). Thus, *STIM1* knockdown causes a small but statistically significant reduction in basal [Ca^2+^]. Reduced basal cytosolic Ca^2+^ was earlier reported after *STIM1* knockdown in HeLa cells (Brandman et al., 2007) and in HUVEC cells (Tsai et al., 2014).

### Transcriptional Profiling of *STIM1* Knockdown NPCs

**FIGURE 3:**
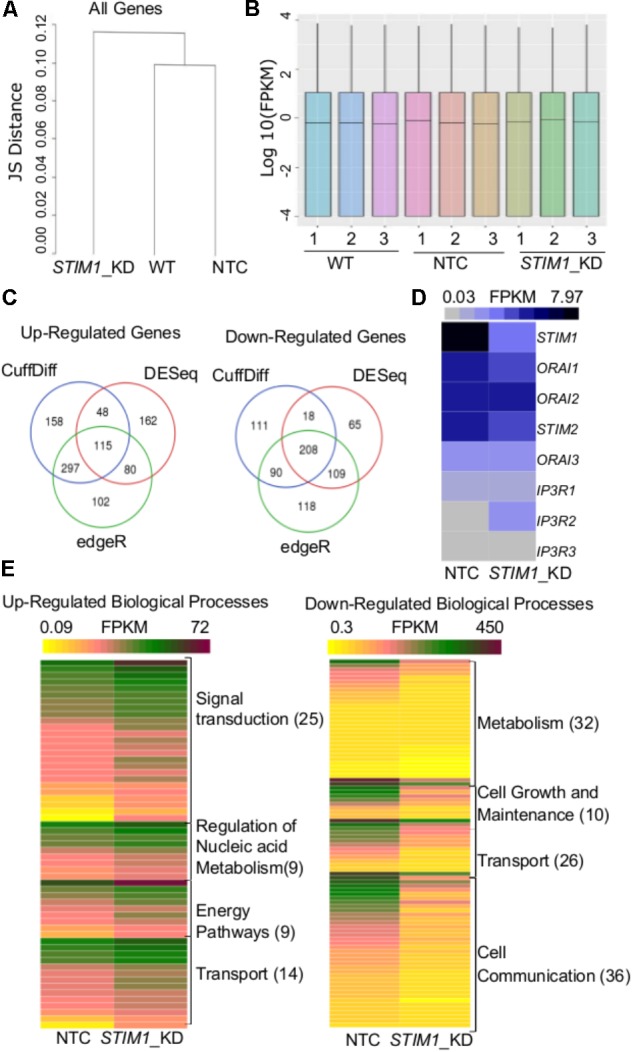
Transcriptome analysis reveals global level changes in NPCs on *STIM1* knockdown. **(A)** A dendrogram of Jensen–Shannon divergences analyzing the pattern of gene expression between wild type, NTC and *STIM1* knockdown NPCs. Hierarchical clustering showing the *STIM1* knockdown cells to form a separate cluster. **(B)** Box plots indicating the distribution of reads across all the samples sequenced. **(C)** Venn Diagrams representing the number of up and downregulated genes in the *STIM1* knockdown NPCs. Genes were tested for differential expression according to Cuffdiff (blue), DESeq (red), and edgeR (green), intersection of genes that were considered differentially transcribed in comparison to control cells were used for further analysis. **(D)** Normalized read counts of the differentially expressed genes involved in SOCE in NTC and *STIM1* knockdown conditions represented as a heat map; FPKM – Fragments Per Kilobase per Million reads (^∗∗^*p* = 0.006; two-tailed *t* -test). **(E)** Functional gene enrichment analysis performed in FunRich with genes in the intersection (115 upregulated and 208 downregulated) showing biological processes which are differentially regulated in the *STIM1* knockdown NPCs based on FPKM values. The number in parentheses represents the number of genes associated with each process in the data set. Three biological replicates per condition were run for RNA-Seq.

The regulation of cell-specific gene expression by SOCE has been described in both non-excitable (Feske et al., 2001, 2005 ; Gwack et al., 2007) and neuronal cells (Mao et al., 2007 ; Lalonde et al., 2014 ; Somasundaram et al., 2014 ; Pathak et al., 2015 ; Richhariya et al., 2017). To identify potential novel gene expression changes by *STIM1* knockdown in human NPCs, we performed parallel genome-wide analysis of mRNA expression profiles in non-transduced NPCs, NTC and the *STIM1* knockdown NPCs. Stable knockdown of *STIM1* leads to global transcriptional changes as evident by the clustering together of non-transduced NPCs with the NTC, whereas the *STIM1* knockdown formed a separate cluster with the Jensen-Shannon divergence as a metric (**Figure 3A**). The distribution of reads was uniform in all samples (**Figure 3B**). Three independent methods, CuffDiff (Trapnell et al., 2012), EdgeR (Robinson et al., 2010), and DESeq (Anders and Huber, 2010) were used for differential expression analysis. Amongst the differentially expressed genes (DEGs) in *STIM1* knockdown NPCs, we further analyzed 115 up-regulated and 208 down-regulated genes, identified by their overlap from all three methods (**Figure 3C**). To understand if *STIM1* knockdown modulates expression of *STIM2* and the SOCE channel *Orai*, we looked at the FPKM values of these genes and confirmed that *STIM1* was the only gene that was significantly down-regulated (**Figure 3D**). Furthermore, we evaluated expression levels of the IP_3_R sub-types because STIMs and the IP_3_Rs are both located on the ER (Béliveau et al., 2014 ; Thillaiappan et al., 2017), where IP_3_R-mediated Ca^2+^-release leads to STIM activation. Transcript levels of three IP_3_R isoforms too did not change significantly in *STIM1* knockdown NPCs (**Figure 3D**). Hence the altered gene profiles of the *STIM1* knockdown hNPCs were not associated with two other classes of SOCE-related genes.

**FIGURE 4:**
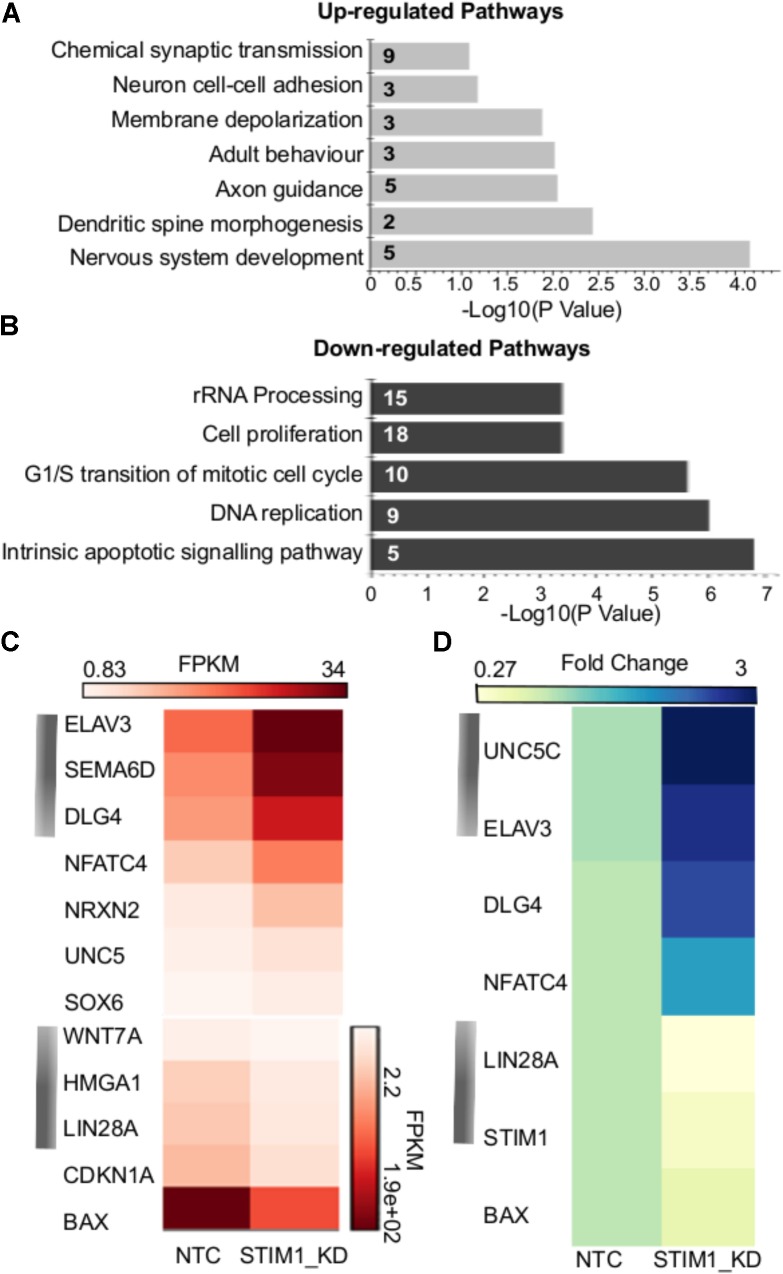
Biological pathways affected by *STIM1* knockdown in NPCs. Genes that were differentially expressed between NPCs with or without *STIM1* knockdown were identified using an enrichment analysis using the DAVID web server. **(A)** Gene-GO term enrichment analysis by DAVID highlighting the most relevant up-regulated biological pathways based on the gene IDs, Each bar represents the Fisher Exact *P* -Value associated with the corresponding enriched pathway and the number in each bar denotes the number of genes involved in each pathway. **(B)** GO terms downregulated in the *STIM1* knockdown NPCs based on the gene IDs. Each bar represents the Fisher Exact *P* -Value associated with the corresponding enriched pathway and the number in each bar denotes the number of genes involved in each pathway. **(C)** Heat map representing normalized read counts of some of the differentially expressed genes in the control and *STIM1* knockdown NPCs. **(D)** Heat map representing fold changes of the indicated genes, as validated by qPCR (*p* < 0.05). Four independent samples were used for validation of the RNA-Seq using RT-PCR. FPKM, Fragments Per Kilobase per Million reads.

The nature of biological processes that might be affected by *STIM1* knockdown was predicted next by analysis of the DEGs. Up-regulated genes associated with biological processes such as signal transduction, regulation of nucleic acid metabolism and energy pathways, whereas down-regulated genes clustered with metabolism, cell growth and maintenance, and cell communication (**Figure 3E**). Genes regulating cellular transport were both up- and down-regulated (**Figure 3E**). The down-regulated processes appeared consistent with a less proliferative state, whereas the up-regulated processes suggested increased cellular specialization and differentiation (Karsten et al., 2003). To understand the nature of signaling mechanisms regulated by *STIM1* in hNPCS, we used DAVID to assess the Gene Ontology (GO) of DEGs. Biological pathways that were significantly up-regulated in *STIM1* knockdown NPCs relative to control NTCs appeared consistent with neuronal differentiation and included nervous system development (GO:0007399), membrane depolarization (GO:0051899), neuron cell-cell adhesion (GO:0007158) and chemical synaptic transmission (GO:0007268) (**Figure 4A**). Conversely, significantly down-regulated pathways in *STIM1* knockdown NPCs suggested reduced cell proliferation and included rRNA processing (GO:0006364), cell proliferation (GO:0008283), G1/S transition of mitotic cell cycle (GO:0000082) and DNA replication (GO:0006260) (**Figure 4B**) (**Table 1**). Intrinsic apoptotic signaling pathway in response to DNA damage by p53 class mediator (GO: 0042771) (**Figure 4B**) was also found to be significantly down-regulated suggesting that *STIM1* knockdown NPCs do not undergo apoptosis.

**Table 1:** Biological pathways enriched by DAVID in *STIM1* KD hNPCs.

Go term	Pathway	*P* -value	Fold enrichment	Bonferroni	Benjamini	FDR	Genes
**Upregulated**							
GO:0007268	Chemical synaptic transmission	6.96E-05	6.491	0.038	0.019	0.101	NRXN2, KIF5A, NPTX2, GRIK4, DLG4, CHRNA4, PRKCG, CACNB3, CACNA1B
GO:0007158	Neuron cell-cell adhesion	0.003	32.458	0.876	0.407	5.265	NRXN2, NLGN4X, ASTN1
GO:0051899	Membrane depolarization	0.008	20.773	0.993	0.637	12.289	CHRNA4, CACNB3, CACNA1B
GO:0030534	Adult behavior	0.009	19.974	0.995	0.598	13.201	NRXN2, NLGN4X, SHANK1
GO:0007411	Axon guidance	0.013	5.443	0.999	0.656	17.590	KIF5A, NGFR, UNC5C, CHL1, SLIT3
GO:0060997	Dendritic spine morphogenesis	#0.066	28.852	1.0	0.950	63.527	DLG4, SHANK1
GO:0007399	Nervous system development	#0.082	3.015	1.0	0.933	71.593	IGSF8, CPLX2, DLG4, SPOCK1, ELAVL3
**Downregulated**							
GO:0006364	rRNA processing	1.62E-07	6.130	2.21E-04	2.21E-04	2.66E-04	EMG1, PNO1, EXOSC5, RPS27L, DIEXF, MRTO4, NOP14, EBNA1BP2, PA2G4, DKC1, DHX37, DDX21, PES1, LTV1, WDR43
GO:0008283	Cell proliferation	1.00E-06	4.301	0.001	6.82E-04	0.001	POLR3G, TP53, CD70, MCM10, PRDX1, CDC25A, PLCE1, PA2G4, DKC1, ASCC3, FRAT2, TXNRD1, NRG1, LRP2, PES1, MYC, EMP1, GNL3
GO:0000082	G1/S transition of mitotic cell cycle	2.43E-06	8.574	0.003	0.001	0.003	CCNE1, CDC6, CDC45, CDKN1A, RRM2, ID4, CDK6, RCC1, MCM10, CDC25A
GO:0006260	DNA replication	4.01E-04	5.078	0.420	0.127	0.655	EXO1, CDC6, CDC45, POLE3, RRM2, MCM10, C10ORF2, CDC25A, DSCC1
GO:0042771	Intrinsic apoptotic signaling pathway in response to DNA damage by p53 class mediator	4.01E-04	14.106	0.420	0.103	0.656	CDKN1A, AEN, TP53, RPS27L, PHLDA3


*Top biological pathways up- and down-regulated in STIM1 KD NPCs vs. control cells. Fisher Exact *P* -values are shown and GO terms are arranged according to their FDR value (False Discovery Rates). All over-represented pathways had a fold change > 2. Both Benjamini–Hochberg and Bonferroni multiple testing correction methods for the occurrence of false positive identifications by adjusting p-values are given. Shown are the gene lists identified in our data set and associated with each pathway. # Indicates *p* -value > 0.05.*

Gene expression changes of a few candidate genes are depicted in **Figure 4C** and some of these were chosen for further validation by quantitative PCR (**Figure 4D**). Markers of neuronal specification such as *Unc5c* (Kennedy et al., 1994), *Elavl3* (Ince-Dunn et al., 2012) and *Dlg4* (also known as the post-synaptic density protein 95, Feng and Zhang, 2009) were up-regulated more than 2.5 fold in *STIM1* knockdown NPCs (**Figure 4D** and data not shown). *Lin28a* and *Bax* were downregulated significantly with a fold change of 5.0 and 1.6 in the *STIM1* knockdown samples (**Figure 4D**, data not shown). Lin28 is an RNA-binding protein enriched in early NPCs and its expression declines during neural differentiation (Yang et al., 2015). Interestingly, Bax initially identified as a pro-apoptotic member of the Bcl-2 family is also shown to regulate Ca^2+^ efflux from the ER, thus influencing Ca^2+^-mediated apoptosis (Scorrano et al., 2003). These data support the hypothesis that *STIM1* knockdown in the NPCs reduces their proliferative and self-renewal capacities and concomitantly induces premature neural differentiation.

### *STIM1* Knockdown Leads to Decreased Proliferation and Early Differentiation of NPCs

**FIGURE 5:**
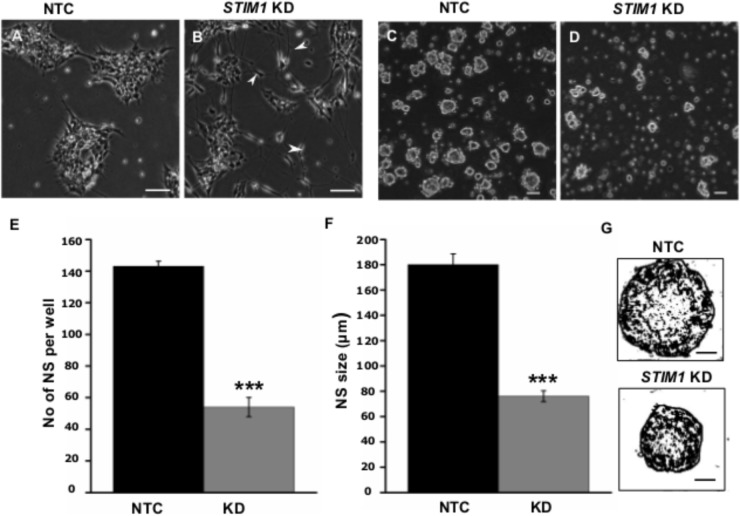
*STIM1* knockdown represses proliferation of NPCs. **(A)** Control (NTC transduced) cells expressing ZsGreen. **(B)** *STIM1* knockdown NPCs undergo spontaneous differentiation as evident by the presence of neurite-like processes and branches. **(C,D)** Neurosphere forming assay (NSA) of NTC and *STIM1* knockdown NPCs at 48 h. **(E)** Quantification of neurosphere (NS) numbers per well after a week of seeding NTC and *STIM1* knockdown NPCs (*n* = 3, *p* = 0.0008). **(F)** Quantification of NS size in microns (μm) at day 7 (*n* = 4, *p* = 0.00067). **(G)** Skeletonized (ImajeJ) NS to show the size difference at day 7 of representative NTC and *STIM1* knockdown NPCs. Scale bar-50 μm **(A–D)**, 20 μm **(G)**.

Based on analysis of the RNA-Seq data, we examined the morphology and proliferative potential of *STIM1* knockdown NPCs. *STIM1* knockdown cells exhibited rapid spontaneous differentiation evident as branched neurites and sparse cell clustering. The control NTC cells, however, resembled wild-type NPCs (compare **Figures 5A,B**). Their growth rates were similar to that of wild type cells (∼24 h population doubling time, passaged every 3–4 days). In contrast, *STIM1* knockdown NPCs cultures took much longer (>7 days) to become confluent and exhibited slower growth as compared with NTCs (**Supplementary Figures S2A,B**). Reduced confluence of *STIM1* knockdown cells might arise from their premature commitment to a more differentiated phenotype and consequently a reduction in the number of undifferentiated NPCs, that are required for repopulating the culture. To obtain a measure of the self-renewal capacity of *STIM1* knockdown cells as compared to NTCs, both were tested by a neurosphere formation assay (Reynolds and Weiss, 1992 ; Pacey et al., 2006). Neural stem cells are known to continuously divide in culture to generate non-adherent spherical clusters of cells, commonly referred to as neurospheres when appropriate plating densities are established (Bez et al., 2003). Differences in the clonal-proliferation ability of *STIM1* knockdown cells were seen as early as 24 h, when very few cell-clusters were visible as compared to the NTCs (data not shown). At 48 h neurospheres were visible in both NTCs and *STIM1* knockdown cultures; however, neurosphere size was greatly reduced in the *STIM1* knockdown condition (compare **Figure 5D** with **Figure 5C**). This impaired proliferation was quantified by counting neurospheres generated after a week in culture. Neurosphere numbers were reduced to less than half of the NTC number in the *STIM1* knockdown cells (**Figure 5E**). Moreover, neurospheres that formed in the NTC cultures were larger in size (180.0 ± 8.3 μm), irrespective of the general heterogeneity in sphere sizes across cultures, as compared with neurospheres in *STIM1* knockdown cultures (76.0 ± 4.32 μm) (**Figures 5F,G**). The percentage of bigger spheres also appeared reduced in *STIM1* knockdown cells. Very small spheres (<50 μm) in both conditions were not scored. It is evident from these experiments that similar to mouse embryonic and adult NPCs (Somasundaram et al., 2014) the clonogenic and proliferative capacities of human NPCs are impaired upon *STIM1* knockdown.

**FIGURE 6:**
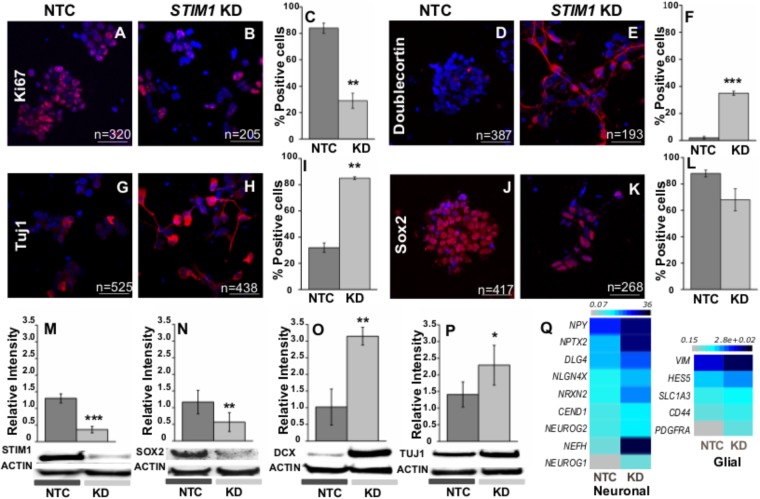
*STIM1* knockdown in NPCs promotes early neural differentiation. Immunostaining and western blot analysis of multipotent and differentiation markers. The cells were counterstained with DAPI for counting the immunopositive cells. **(A,B)** Immunostaining of the control and *STIM1* knockdown NPCs for the proliferation marker Ki-67 and **(C)** its quantification as shown in the graph (*p* = 0.0043). **(D,E)** Expression of Doublecortin (DCX) a marker of newly born neurons in the NTC and *STIM1* knockdown NPCs and **(F)** its quantification as shown in the graph (*p* = 1.67 × 10^-4^). **(G,H)** Neuron-specific Class III β-tubulin (Tuj1) in the NTC and *STIM1* knockdown NPCs and **(I)** its quantification as shown in the graph (*p* = 0.0087). **(J,K)** Sox2, the multipotent neural stem cell marker the NTC and *STIM1* knockdown NPCs and **(L)** its quantification as shown in the graph, not significant. Scale bar-50 μm. Total number of cells counted (n) as indicated in each panel. Western blot analysis showing **(M)** STIM1, *p* = 0.0006 **(N)** Sox2, *p* = 0.008 **(O)** DCX, *p* = 0.0056 **(P)** Tuj1 protein (*p* = 0.043) levels in the control and knockdown cells. **(Q)** Heat map representing normalized read counts of selected neuronal and glial genes which are upregulated in the *STIM1* knockdown NPCs. *N* ≥ 3, *t* -test used for all significance tests. Asterisks indicate ^∗∗∗^*p* < 0.001, ^∗∗^*p* < 0.01, ^∗^*p* < 0.05.

Premature differentiation and the reduced proliferative potential of *STIM1* knockdown NPCs were further assessed by immunostaining with appropriate markers. The nuclear Ki-67 protein (pKi-67) has been shown to express exclusively in proliferating cells (Scholzen and Gerdes, 2000). More than 80% of control cells (NTC) were positive for Ki67 suggesting that they are dividing actively, whereas in *STIM1* knockdown cells just 30% were positive for Ki67 (**Figures 6A–C**). *PCNA* (proliferating cell nuclear antigen), another marker for proliferation in eukaryotic cells (Strzalka and Ziemienowicz, 2011) was also significantly down-regulated in the *STIM1* knockdown cells as evident from the RNA-Seq data (Fold change -1.4, *p* = 0.03, GSE109111). In parallel with reduced expression of proliferation markers, several proteins related to neural differentiation were up-regulated in *STIM1* knockdown NPCs, where knockdown of STIM1 protein was re-confirmed (**Figure 6M** and **Supplementary Figure S3A**). Expression of differentiation markers in the *STIM1* knockdown cells was analyzed within 7 days of culture. As in NTCs, we did not observe changes in the numbers of glial progeny. However, the ability of *STIM1* knockdown in NPCs, to alter glial fate and numbers could manifest at later stages of differentiation. This has not been tested.

Doublecortin (DCX) is a brain-specific microtubule-associated protein that regulates neuronal migration and is associated with neurogenesis (Francis et al., 1999). DCX levels were negligible in control cells whereas 35% of *STIM1* knockdown cells expressed DCX (**Figures 6D–F**). Expression of DCX was further confirmed in *STIM1* knockdown cells by western blots (**Figure 6O** and **Supplementary Figure S3C**). Immunoreactivity of neuronal βIII Tubulin, Tuj1 was also higher in *STIM1* knockdown cells (85.0% ± 0.9) as compared to NTCs (∼35%). Moreover, *STIM1* knockdown NPCs expressing TUJ1 exhibit branched neurites, unlike the NTCs, where such neuronal morphology was not evident (**Figures 6G–I,P** and **Supplementary Figure S3D**). Expression of Sox2, a transcription factor required in part for the maintenance of NPC properties and functions, through the Shh and Notch pathways (Wegner, 2011) was tested next in *STIM1* knockdown NPCs. It is known that deletion of Sox2 in the mouse attenuates the self-renewal capacity of hippocampal NSCs (Favaro et al., 2009). Sox2-positive cells were somewhat lower in number in the *STIM1* knockdown cells (**Figures 6J–L**) and total Sox2 protein as measured by western blots was significantly reduced in *STIM1* knockdown NPCs (**Figure 6N** and **Supplementary Figure S3B**). Thus, *STIM1* knockdown in human NPCs induces early neural differentiation that would eventually deplete the NPC pool. Indeed, transcript levels of many neuronal (*NPY, NPTX2, DLG4, NLGN4X, NRXN2, CEND1, NEFH, NEUROG2, NEUROG1*) and some early glial markers (*HES5, SLC1A3, CD44, PDGFRA*) were also significantly up-regulated in the *STIM1* knockdown NPCs as evident from RNA-Seq data (**Figure 6Q**, GSE109111). Physiologically NPCs/NSCs need to fine-tune quiescence and proliferation/commitment to guarantee lifelong neurogenesis and avoid premature exhaustion (Cavallucci et al., 2016). Knock-down of *STIM1* appears to tip this balance and push the cells toward a differentiated phenotype.

## Discussion

The ability of human neural precursor cells to maintain their proliferative potential and generate neurons or glia in a spatiotemporal manner is important in the context of multiple neurological and psychiatric disease conditions (Ishibashi et al., 1995 ; Elder et al., 2006 ; Shi et al., 2008 ; Cramer et al., 2011). In this study we have identified the ER Ca^2+^ sensor STIM1 as essential for Store Operated Calcium Entry (SOCE) in human NPCs. Global gene expression changes were observed in hNPCs after a stable knockdown of *STIM1* that is likely a consequence of reduced SOCE. STIM1 knockdown in hNPCs reduced the expression of genes associated with cell proliferation and upregulated genes belonging to pathways for nervous system development and differentiation. Furthermore, morphological and immunohistochemical analyses identified a fate change in *STIM1* knockdown NPCs where the balance between self-renewal and differentiation was altered significantly toward the latter. Mouse neural precursors with loss of function of the SOCE channel Orai exhibit very similar fate changes (Prakriya et al., 2006 ; Lewis, 2007 ; Domenichini et al., 2018).

### Role of ER Ca^2+^ and SOCE in Mammalian Neurogenesis

Ca^2+^ signals affect the earliest steps of neurogenesis including neural induction, differentiation of neural progenitors into neurons, and the neuro-glial switch (Leclerc et al., 1997, 2000, 2012). The endoplasmic reticulum (ER) is a major Ca^2+^ storage organelle that contributes to multiple Ca^2+^ signaling pathways. In hESC-derived neural progenitors, Ca^2+^ mobilization from intra- and extracellular compartments occurs in response to depolarization and by activation of glutamatergic and dopaminergic receptors (Malmersjö et al., 2010). In agreement with published data demonstrating purinergic, ATP- and glutamate-induced [Ca^2+^]_i_ responses in hNPCs (Forostyak et al., 2013) RNA-Seq analysis of hNPCs derived in this work confirmed purinergic receptor expression (*P2RX_3_, P2RX_4_, P2RY_1_* and *P2RY_11_*) at FPKM > 3 and robust expression of an ionotropic glutamate receptor (*GRINA* or *NMDARA1* : FPKM > 90). Expression of several subclasses of TRP channels (*TRPC1, TRPC4, TRPM4, TRPM7, TRPV1* (FPKM > 3) was also evident (GSE109111). Interestingly the neural ‘N’ type voltage-gated Ca^2+^ channel *CACNA1B* and the β subunit *CACNB3* present in hNPCs (FPKM > 2) showed significant upregulation in *STIM1* knockdown (GSE109111) possibly as a compensatory mechanism for maintaining cellular Ca^2+^ homeostasis. Upregulation of a TRP channel has been observed in IP_3_ receptor mutant neurons of *Drosophila* where it appears to compensate for changes in cellular calcium homeostasis (Chakraborty and Hasan, 2017). Moreover, Calreticulin, a luminal Ca^2+^ buffering protein in the ER is also regulated upon neural differentiation in mouse ESCs (Wang and Gao, 2005). Thus, stage-specific regulation of ER Ca^2+^-release and SOCE impacts neuronal development across invertebrates and vertebrates and appears essential for neural differentiation of neural precursors in mammals including humans.

The physiological significance of neuronal SOCE in neural precursors requires further investigation particularly in the context of neurological disorders. The RNA-Seq data demonstrate that hNPCs also express several classes of receptor tyrosine kinases belonging primarily to the FGF and EGF receptors families that are known to activate Ca^2+^ signaling through Phospholipase Cγ, some of which are differentially expressed in the *STIM1* knockdown NPCs. The reduced proliferative potential of *STIM1* knockdown NPCs might derive from altered expression of receptor tyrosine kinases (GSE109111). Transcriptional profiling downstream of reduced SOCE, as reported here, is a first step in understanding the cellular and molecular mechanisms underlying SOCE-related human neurological disorders and the use of pluripotent cell-derived NPCs for disease modeling and cell-based therapies (Brafman, 2015).

### *STIM1* Affects Self-Renewal of Human NPCs

Neural stem cells (NSCs) or neural precursor cells (NPCs) (first reported by Altman and Das, 1965) *in vivo* are derived from the nervous system, can self-renew and can give rise to cells other than themselves through asymmetric cell division (Gage, 2000). NPCs require a specific metabolic state to maintain the balance between self-renewal and differentiation and decreased metabolic demand and impaired renewal is associated with various brain disorders (Knobloch et al., 2013; Kim et al., 2014). Proliferative stem cells in mammalian and *Drosophila* nervous systems are highly anabolic. Their high levels of lipogenesis and decreased amino acid and lipid oxidation regulate cell proliferation and determine cell fate (Knobloch et al., 2013; Homem et al., 2014, 2015). SOCE also contributes to metabolic reprogramming of naive T cells by regulating expression of components of the PI3K-AKT kinase-mTOR nutrient-sensing pathway (Vaeth et al., 2017). It is possible that reduced cell metabolism, one of the biological processes, enriched in our study (**Figure 3E**) leads to decreased cell proliferation of the *STIM1* knockdown cells. Knock-down of *STIM1* and *Orai1* also inhibit proliferation of vascular smooth muscle cells (Potier et al., 2009) and knockdown of either *STIM1*, *STIM2* or *Orai1* inhibit endothelial cell proliferation leading to cell cycle arrest at S and G2/M phase (Abdullaev et al., 2008). Several other studies suggest that SOCE and metabolism are interlinked. Astrocytic glycogenolysis in murine cerebellar and cortical cultures is stimulated by SOCE (Muller et al., 2014). In *Drosophila*, altered lipid metabolism has been described in mutants with attenuated Ca^2+^-release through the IP_3_R and reduced SOCE (Subramanian et al., 2013; Baumbach et al., 2014). A cell-intrinsic role of SOCE in regulating lipid metabolism in mice and cells from human patients defective in SOCE has also been shown (Arruda et al., 2017; Maus et al., 2017). Together these data suggest that *STIM1* and SOCE support a high-energy metabolic state in various cells including NPCs, which in turn facilitates cell proliferation.

### Relevance to Neurodevelopmental and Neuropsychiatric Disorders

Mammalian neuroepithelial cells or NPCs *in vivo*, initially undergo symmetric proliferative divisions (Rakic, 1995) to expand their population and later switch to asymmetric divisions that produce more of themselves and a lineage specified downstream cell (Huttner and Kosodo, 2005; Florio and Huttner, 2014). The transition from symmetric to asymmetric division is tightly regulated, and precocious asymmetric divisions result in premature differentiation and smaller brains (Cabernard and Doe, 2009; Gauthier-Fisher et al., 2009; Egger et al., 2010). Aberrant NPC regulation is now linked to many developmental and degenerative disorders of the brain (reviewed by Ladran et al., 2013). The stable knockdown of *STIM1* significantly reduced NPC proliferation concurrently inducing their spontaneous differentiation to neural progenitors. In a systemic context, this phenotype might lead to an early cessation of the stem cell pool in the brain and differentiated neural cell types that are likely to be dysfunctional or exhibit sub-optimal functions. Interestingly patients with *STIM1* mutations in the EF hand or CC domains have been reported recently with psychiatric disturbances (Harris et al., 2017). Moreover, in a large genome-wide analysis of psychiatric patients by Cross-Disorder Group of the Psychiatric Genomics Consortium (2013), dysregulated Ca^2+^ homeostasis was identified in several disorders. In this context, neurodevelopmental aberrations arising from abnormal specification, growth, expansion and differentiation of embryonic NPCs are thought to contribute to neurological disorders that initiate in childhood as well as toward certain adult-onset mental disorders, including autism, language disorders and mental retardation (Stevens et al., 2010). In differentiated neurons, SOCE is thought to regulate neuronal gene expression (Lalonde et al., 2014), and the maturation and maintenance of dendritic spines (Sun et al., 2014; Korkotian et al., 2017). Defective SOCE has been associated with several neurodegenerative disorders, such as Huntington’s disease (Nekrasov et al., 2016; Wu et al., 2011, 2016), Alzheimer’s disease (Leissring et al., 2000; Yoo et al., 2000; Zhang et al., 2015), Parkinson’s disease (Zhou et al., 2016), spongiform encephalopathies (Lazzari et al., 2011) and chorea-acanthocytosis (Pelzl et al., 2017). In the light of our findings in NPCs, it is tempting to speculate that deficient SOCE alters neuronal gene expression patterns in a temporal manner resulting in disease pathologies that manifest over several years. Further investigation of *STIM1* function and SOCE in neurodevelopmental, neurodegenerative and psychiatric disorders could thus generate novel therapeutic insights.

## Author Contributions

RG designed and performed the experiments and wrote the manuscript. BD performed the experiments and edited the manuscript. PC performed the experiments, analyzed the data, and edited the manuscript. GH designed the experiments and wrote and edited the manuscript.

## Conflict of Interest Statement

RG and GH are involved in the provisional patent application that is in progress entitled “Human neural precursor cells with inducible STIM1 knockdown to model neurodevelopmental and neuropsychiatric diseases with altered intracellular calcium signaling”. The remaining authors declare that the research was conducted in the absence of any commercial or financial relationships that could be construed as a potential conflict of interest.

## Supplementary Material

FIGURE S1 Experimental design.(1) Small molecule-derived neural precursor cells (NPCs) were derived from the human embryonic stem cell line (hESC) H9 using a dual smad inhibition protocol with small molecules. (2) NPCs were transduced with *STIM1* shRNA-miR to generate a stable knockdown line. (3) *STIM1* knockdown was validated with Ca^2+^ imaging to confirm the attenuation of store-operated Ca^2+^ entry (SOCE) and western blot for validation of a corresponding decrease in STIM1 protein levels. (4) RNA-Seq analysis was performed to understand the global transcriptional changes subsequent to *STIM1* knockdown in the control NTC and *STIM1* knockdown NPCs. (5) Data analysis after RNA-Seq led to enriched biological pathways which are significantly up/downregulated in the *STIM1* knockdown NPCs. The differentially expressed genes (DEGs) and pathways were taken as cues to conduct proliferation assays (phenotype) and other readouts to corroborate the bioinformatic analyses to the observed morphological changes. The findings were validated at the protein level by immunostaining and western blots. hESC, human embryonic stem cells; hNPCs, human small molecule-derived neural precursor cells; *STIM1* KD hNPCs, *STIM1* knockdown human small molecule-derived neural precursor cells; NTC, non-targeting control (vector control); DEGs, differentially expressed genes.

FIGURE S2 Cell growth assays. **(A)** Growth curve of the control NTC and *STIM1* knockdown NPCs grown as adherent monolayer at 5 DIV in triplicates, *n* = 3. **(B)** MTT assay showing significant decrease in cell growth in the *STIM1* knockdown NPCs when normalized to the NTC control cells, *n* = 5, *p* = 0.008. DMSO was used to solubilize formazan crystals. MTT assay was performed during the log phase of the cell cycle and cellular metabolic activity due to NAD(P)H flux resulting in formazon production was used an indicator of viable cells or proliferation. Cells at corresponding phases of cell cycle were stained separately with trypan blue to rule out apoptosis as a reason of low metabolic activity.

FIGURE S3 Original images of Western blots. NTC, NTC1 and NTC2 represent biological replicates of control NPCs and KD, KD1 and KD2 represent corresponding *STIM1* knockdown NPCs. Molecular weight markers and loading order of lanes in each blot are indicated in each panel. **(A)** Full blots for **Figures 2D**, **6M**. Green box indicates the cropped area shown in **Figure 2D**, red box indicates the cropped area shown in **Figure 6M**. Top panel - STIM1 (85 KDa), bottom panel - Actin (42 KDa). **(B)** Red box indicates the cropped area shown in **Figure 6N**. Top panel - Sox2 (35 KDa), bottom panel - Actin. Due to similar molecular weights Sox2 and Actin were run on adjacent lanes in the same gel. **(C)** Red box indicates the cropped area shown in **Figure 6O**. Left panel - Doublecortin (45 KDa), right panel - Actin. Due to similar molecular weights Doublecortin and Actin were run on adjacent lanes in the same gel. **(D)** Red box indicates the cropped area shown in **Figure 6P**. Left panel - Tuj1 (55 KDa), right panel – Actin. The same blot is shown at different exposures.
