# Supplementary figures and images for: Stable STIM1 Knockdown in Self-Renewing Human Neural Precursors Promotes Premature Neural Differentiation
Source: Front Mol Neurosci. 2018 Jun 11;11:178. doi: 10.3389/fnmol.2018.00178 (PMC6004407; doi:10.3389/fnmol.2018.00178)

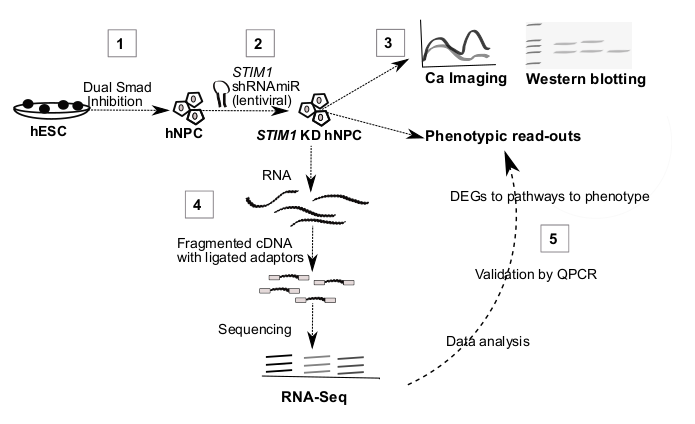

Supplement: FIGURE S1 — Experimental design.(1) Small molecule-derived neural precursor cells (NPCs) were derived from the human embryonic stem cell line (hESC) H9 using a dual smad inhibition protocol with small molecules. (2) NPCs were transduced with STIM1 shRNA-miR to generate a stable knockdown line. (3) STIM1 knockdown was validated with Ca2+ imaging to confirm the attenuation of store-operated Ca2+ entry (SOCE) and western blot for validation of a corresponding decrease in STIM1 protein levels. (4) RNA-Seq analysis was performed to understand the global transcriptional changes subsequent to STIM1 knockdown in the control NTC and STIM1 knockdown NPCs. (5) Data analysis after RNA-Seq led to enriched biological pathways which are significantly up/downregulated in the STIM1 knockdown NPCs. The differentially expressed genes (DEGs) and pathways were taken as cues to conduct proliferation assays (phenotype) and other readouts to corroborate the bioinformatic analyses to the observed morphological changes. The findings were validated at the protein level by immunostaining and western blots. hESC, human embryonic stem cells; hNPCs, human small molecule-derived neural precursor cells; STIM1 KD hNPCs, STIM1 knockdown human small molecule-derived neural precursor cells; NTC, non-targeting control (vector control); DEGs, differentially expressed genes. [file Image_1.TIFF]

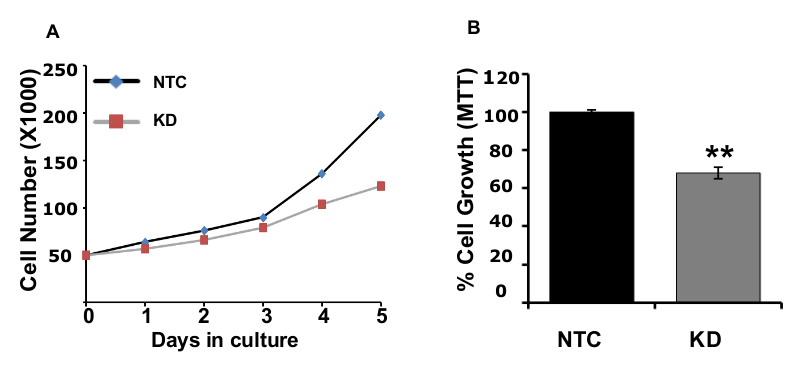

Supplement: FIGURE S2 — Cell growth assays. (A) Growth curve of the control NTC and STIM1 knockdown NPCs grown as adherent monolayer at 5 DIV in triplicates, n = 3. (B) MTT assay showing significant decrease in cell growth in the STIM1 knockdown NPCs when normalized to the NTC control cells, n = 5, p = 0.008. DMSO was used to solubilize formazan crystals. MTT assay was performed during the log phase of the cell cycle and cellular metabolic activity due to NAD(P)H flux resulting in formazon production was used an indicator of viable cells or proliferation. Cells at corresponding phases of cell cycle were stained separately with trypan blue to rule out apoptosis as a reason of low metabolic activity. [file Image_2.TIFF]

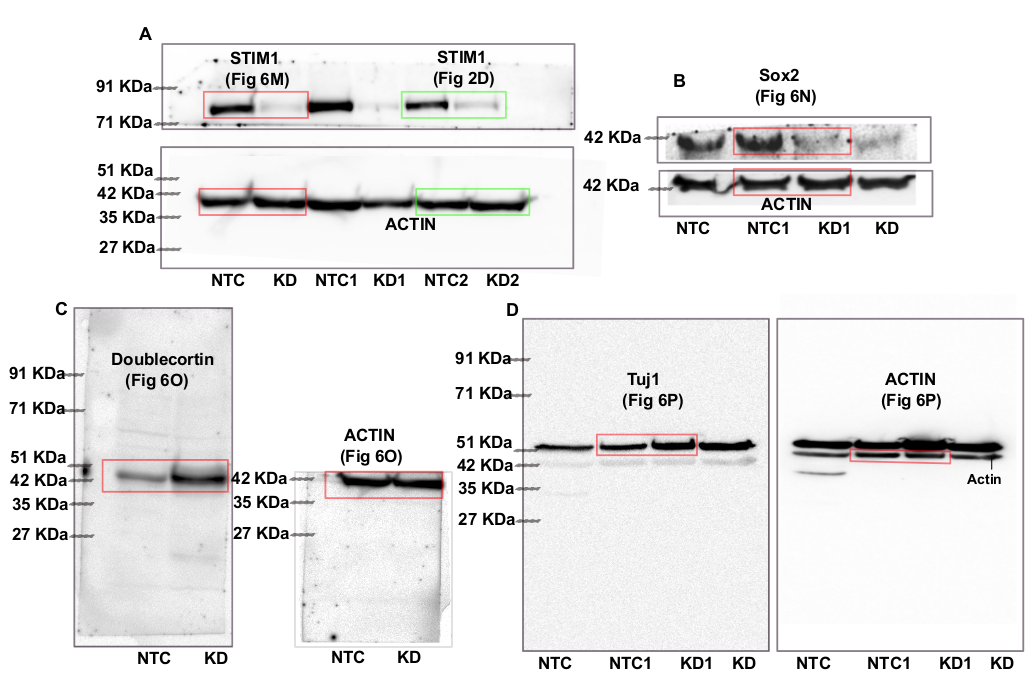

Supplement: FIGURE S3 — Original images of Western blots. NTC, NTC1 and NTC2 represent biological replicates of control NPCs and KD, KD1 and KD2 represent corresponding STIM1 knockdown NPCs. Molecular weight markers and loading order of lanes in each blot are indicated in each panel. (A) Full blots for Figures 2D, 6M. Green box indicates the cropped area shown in Figure 2D, red box indicates the cropped area shown in Figure 6M. Top panel - STIM1 (85 KDa), bottom panel - Actin (42 KDa). (B) Red box indicates the cropped area shown in Figure 6N. Top panel - Sox2 (35 KDa), bottom panel - Actin. Due to similar molecular weights Sox2 and Actin were run on adjacent lanes in the same gel. (C) Red box indicates the cropped area shown in Figure 6O. Left panel - Doublecortin (45 KDa), right panel - Actin. Due to similar molecular weights Doublecortin and Actin were run on adjacent lanes in the same gel. (D) Red box indicates the cropped area shown in Figure 6P. Left panel - Tuj1 (55 KDa), right panel – Actin. The same blot is shown at different exposures. [file Image_3.TIFF]
